# A Narrative Review of the Impact of Sleep on Athletes: Sleep Restriction Causes and Consequences, Monitoring, and Interventions

**DOI:** 10.7759/cureus.76635

**Published:** 2024-12-30

**Authors:** Madjer Hatia, Nuno Loureiro, Júlia Ribeiro, Frederico Moeda, Manuel Melo, João Tocha, Afonso Schonenberger, Cláudia Correia

**Affiliations:** 1 Physical Medicine and Rehabilitation, Unidade de Saúde Local de Lisboa Ocidental, Lisboa, PRT; 2 Sports Medicine, Sporting Clube de Portugal, Lisboa, PRT; 3 Physical Medicine and Rehabilitation, Hospital de Faro, Faro, PRT

**Keywords:** athletes, inadequate sleep, interventions, monitorization, performance, sleep

## Abstract

This narrative review synthesizes evidence on the impact of sleep on athletic performance, the prevalence and causes of sleep disturbances, and effective monitoring and intervention strategies to enhance sleep quality and duration. A comprehensive review of case studies, observational studies, randomized controlled trials, systematic reviews, and meta-analyses was conducted using PubMed, Cochrane Library, and Google Scholar up to July 2024. Sleep plays a crucial role in the overall well-being and performance of athletes, yet sleep issues are highly prevalent due to factors such as competition schedules, psychological stress, and travel across time zones. Inadequate sleep negatively affects physical strength, endurance, cognitive function, and injury risk. Sleep tracking is highlighted as both a diagnostic and therapeutic tool, with interventions such as extending sleep duration, adopting sleep hygiene practices, and, when necessary, pharmacological approaches proving effective. The review concludes that tailored sleep strategies are essential to optimize athletic performance, recovery, and overall well-being.

## Introduction and background

Sleep is a crucial human behavior that significantly impacts the proper development of our biology, psychology, and social well-being. It also has both immediate and long-term effects on physical, mental, and cognitive health [1].

A typical seven- to eight-hour sleep period consists of five sleep cycles, each lasting approximately 90 minutes [2]. Each sleep cycle consists of several distinct phases: Stage 1, lasting 5-10 minutes, is characterized by the brain generating slow waves (SW) called theta waves. Stage 2 is a relatively light sleep. Stages 3 and 4 are referred to as deep sleep or a state of being completely unaware of the surroundings. Stage 5 is rapid eye movement (REM) sleep, during which the sleeper is on the verge of consciousness and likely experiencing dreams. These stages are deemed essential for the process of physical and psychological restoration [2].

There is an assumption that athletes may need more sleep than nonactive individuals to properly recover and adapt between exercise sessions. It is proposed that athletes may require about 9 or 10 hours of sleep, compared to the regular recommendations of seven to nine hours for adults. However, studies consistently report average sleep durations of less than 8 hours per night among athletes across age groups and disciplines [3].

Recently, there has been a growing focus on the importance of sleep for athletic performance, cognition, health, and mental well-being [4].

An increasing amount of data indicates that inadequate sleep (1) may affect muscle strength, speed, and other components of physical performance; (2) is a risk factor for injuries; (3) can impair recovery following injury; (4) increase the risk of concussions; (5) affect cognitive performance, such as alertness, learning and memory, decision-making, and creativity; and (6) affect mental health, which is vital not only for athletic performance but also for the overall well-being of athletes [1,3-4,5-10].

Sleep restriction (SR) refers to a disruption in the regular sleep-wake cycle where individuals either go to bed later or wake up earlier than usual. On the other hand, sleep deprivation (SD) typically refers to severe instances of sleep deficiency, where individuals go without any sleep for an extended duration, such as entire nights [1].

Epidemiology

According to Charest and Grandner, the average sleep length in elite athletes, calculated by actigraphy, was around 6.5 hours; 39.1% of athletes self-reported having inadequate sleep, defined as less than seven hours, and the average number of nights per week in which athletes felt they did not get sufficient sleep was 3.8 [4]. Additionally, there is frequent substandard sleep quality among athletes, regardless of the specific sport. The quality of sleep was assessed through the Pittsburgh Sleep Quality Index (PSQI), and 19.8% to 50% of the participants exhibited a score >5 (suggesting poor quality of sleep) [4]. The high prevalence of daytime sleepiness, ranging from 32.7% to 62.9%, especially in young athletes, was measured by self-assessment and the Epworth Sleepiness Scale (score ≥10). [4] The literature consistently reported a high prevalence of poor sleep quality, and the most common symptoms were fatigue, lethargy, drowsiness, and inability to stay awake [1,4].

Halson and Juliff revealed disparities in sleep and waking patterns between training and no training days. Before training days, the time spent in bed was considerably shorter, and the amount of sleep achieved was significantly less than on nights before rest days. It was also observed that athletes who participate in team sports sleep an additional 30 minutes compared to athletes who participate in individual sports [6].

## Review

Importance of sleep

In elite athletes, a strong correlation exists between getting sufficient sleep quality and experiencing better athletic performance, including higher accuracy, faster reaction times, increased physical endurance, improved cognitive performance, and psychological resilience [6,11]. Prior research in team sports has shown that success in competition is correlated with longer and higher quality sleep [3].

Quality sleep after intense physical activity provides restorative advantages and facilitates a quicker recovery [11]. The 24-hour plasma growth hormone (GH) profile in healthy young individuals typically exhibits low baseline levels, periodically marked by abrupt secretion surges. In healthy women, daytime GH secretion occurs frequently in pulses, but in men, a pulse linked with sleep onset is usually the predominant or sole daily instance of active secretion. Substantial evidence demonstrates a reliable correlation between SW sleep and elevated GH secretion [12]. Adequate sleep provides higher levels of GH that facilitate muscle tissue growth and restoration, speeding up recovery [11]. Sleep problems increase overall injury risk, and this relationship is mostly studied regarding concussions [1].

Causes of sleep problems

Factors associated with professional athletic competition that can worsen sleep health include (1) the timing of competitions, (2) congested match or game schedules, (3) traveling, (4) specific medical issues, and (5) psychological anguish related to the competition or other factors (Table 1).

**Table 1:** Causes of sleep problems Factors contributing to poor sleep in athletes include physical, psychological, and environmental causes that disrupt sleep patterns and quality.

Cause	Description	References
Physical stress and injuries	Muscle soreness and hormonal imbalances from intense training interfere with sleep.	Cook and Charest, 2023 [1]
Travel across time zones	Circadian disruption and fatigue from travel reduce sleep quality and duration.	Charest and Grandner, 2020 [4]
Competition timing	Late competitions increase arousal and core body temperature, delaying sleep onset.	Halson and Juliff, 2017 [6]
Congested schedules	Frequent matches and post-competition obligations (e.g., media, injury care) limit time for sleep.	Halson and Juliff, 2017 [6]
Medical issues	Conditions like sleep-disordered breathing and restless legs syndrome are more prevalent in athletes due to physical stress and body composition.	Nobari et al., 2023 [2]
Caffeine and diet	Caffeine intake and high-calorie diets can disrupt sleep patterns.	Watson, 2017 [3]
Psychological factors	Anxiety, stress, and excitement from competition lead to pre-sleep hyperarousal and insomnia.	Watson, 2017 [3]
Use of electronic devices	Increased screen time before bed delays melatonin release and disrupts circadian rhythms.	Watson, 2017 [3]

Delayed sleep onset time may be caused by later competition timings that increase arousal and elevate core body temperature at night, making it harder to fall asleep. Exposure to bright light, particularly during nighttime competitions, can disrupt an athlete's circadian rhythm. Caffeine consumption may have decreased both the quality and amount of sleep. Post-competition obligations, such as team gatherings, media obligations, injury care, and traveling, may also lead to inadequate sleep [11].

Cook and Charest identified that training load and schedule could disrupt the sleep quality of elite athletes due to heightened physical exhaustion, earlier waking hours, heightened stimulation, and raised internal body heat [1,6].

Traveling across time zones leads to declining sleep health due to circadian disruption, travel fatigue, and irregular sleep practices. Research demonstrates connections between traveling across different time zones and the resulting performance outcomes [1,3,6].

Athletes may be more susceptible to certain sleep-related medical issues due to physical stress, increased muscle bulk, and overtraining, which can disrupt hormonal balance and sleep quality. High-calorie diets or increased body mass, especially in certain sports, may narrow airways and elevate sleep-disordered breathing (SDB) risk, while iron deficiency common in endurance athletes can trigger restless legs syndrome (RLS). Additionally, frequent travel and schedule disruptions can disturb circadian rhythms, and heightened nervous system activity from intense training or competition may exacerbate sleep issues. Addressing recovery, diet, and training balance can help mitigate these risks. While SDB is believed to impact only 4% of the general population, it is present in 14% of professional football players. RLS is increasingly acknowledged as a significant factor contributing to sleep disturbances, particularly among athletes, which was not fully understood [3].

Before the competition, lack of sleep might be attributed to the presence of intense psychological stress, anxiety, and excitement experienced by athletes during competition, which can increase pre-sleep hyperarousal and contribute to insomnia [11].

After a competition, increased levels of adrenaline and cortisol following stressful situations are linked to increased alertness before sleep and engaging in repetitive thoughts about one's performance, which can result in a delay in the time one falls asleep later, especially after experiencing losses [11].

Due to simultaneous academic stress, young and college athletes may also have additional challenges with the length and quality of their sleep. Frequently, this implies that athletes must relinquish sleep to adequately balance their academic and sports obligations, making younger athletes more susceptible to SD [3].

Additional factors that may contribute to the physical well-being of professional athletes include the frequency and severity of physical injuries and illnesses, the timing of electronic device usage, and dietary habits such as meal timing and composition. While these factors are not exclusive to professional athletes, they are more prevalent in this group due to their demanding lifestyles and schedules [1]. Alcohol consumption post-competitions occurs as a kind of reward or as a customary practice in sports culture (e.g., the post-match gathering). This behavior has been linked to a decrease in the duration of REM sleep and an increase in sleep disturbances [11].

Consequences of lack of sleep

Performance

SR decreases athletic performance by decreasing endurance, muscular strength, and power [4,5]. After SR, athletes become exhausted more quickly than expected (Table 2) [4].

**Table 2:** Consequences of lack of sleep The physiological, cognitive, and performance-related impacts of inadequate sleep on athletes highlight its role in injury risk and recovery.

Consequence	Description	References
Increased injury risk	Greater susceptibility to injuries, particularly in athletes with sleep debt or chronic sleep restriction.	Copenhaver and Diamond, 2017 [5]
Mental health issues	Elevated risk of depression, anxiety, and poor psychological resilience.	Halson and Juliff, 2017 [6]
Poor immune function	Increased susceptibility to illnesses due to compromised immune response.	Nobari et al., 2023 [2]
Cognitive impairment	Slower reaction times, decreased attentiveness, impaired decision-making, and hindered creative problem-solving.	Watson et al., 2017 [3]
Decreased athletic performance	Reduced endurance, muscular strength, and power output; faster onset of exhaustion.	Craven et al., 2022 [10]
Impaired recovery	Lower growth hormone secretion, increased cortisol and inflammatory markers, impaired muscle repair, and glycogen repletion.	Van Cauter and Copinschi, 2000 [12]

Regarding aerobic demands, the studies show a decline in maximal work rate after SR. Furthermore, research in students, football players, and judo athletes has demonstrated that both average and maximum power output during Wingate anaerobic cycle tests declines in individuals who have had a single night of SR of four hours [5].

The limited exercise tolerance following SR is believed to be caused by either the impairment of aerobic pathways or perceptual alterations. Undoubtedly, a rise in perceived effort and a decrease in power production would provide evidence for neuromuscular factors contributing to exhaustion [5].

Risk Factors for Injury

Research has consistently shown that inadequate sleep markedly increases the risk of injury among athletes. A study by Dwivedi et al. reported that adolescent athletes who slept fewer than eight hours per night were 1.7 times more likely to sustain an injury than those achieving eight or more hours of sleep [13]. Similarly, O’Sullivan and Johnston indicated a higher incidence of injuries among athletes sleeping less than seven hours per night [14]. Furthermore, a study by Dwivedi et al. demonstrated that insufficient sleep, defined as less than 8.1 hours per night, significantly elevated the risk of injury [4]. Compounding this issue, the cumulative effect of chronic partial SR, often referred to as sleep debt (the progressive build-up of sleep pressure due to prolonged insufficient sleep), has been identified as a critical factor contributing to injury susceptibility over time [2].

Concussion

Concussion is frequently observed in sports, especially in team sports. Its diagnosis is a challenging task, and it is often unnoticed [4]. In some studies, insomnia and excessive daytime drowsiness were identified as risk factors for concussion, outperforming established risk factors such as participating in high-risk sports or having a history of concussions [4,10].

A recent meta-analysis reported a high incidence (about 50%) of sleep disturbances following a concussion, indicating that these disturbances are likely a consequence of the injury. Disrupted sleep patterns can exacerbate coexisting symptoms such as depression, fatigue, and pain, further impeding the recovery process by interfering with the body's natural healing mechanisms during sleep [4,10]. Additionally, the persistence of these post-concussion sleep disturbances serves as a reliable predictor of a prolonged recovery period [4,10].

Recovery

Disrupted sleep directly impacts the release of GH and cortisol secretion and elevates proinflammatory cytokines, such as interleukin-6 and C-reactive protein. In addition, those who lack sufficient sleep may consume more unhealthy meals, negatively impacting glycogen repletion and protein synthesis. Altogether, this ultimately impacts the immune system and impairs the ability of muscles to recover and repair damage caused by intense training, which may contribute to the development of overtraining in athletes [4].

Mental and Cognitive Performance

Inadequate sleep quality and SD hinder brain functions, impacting many cognitive processes that potentially aid in the restoration of mental exhaustion, either directly or indirectly [4].

Research has shown that SD has a detrimental effect on attentiveness and reaction time and can impact an athlete's capacity to quickly and accurately choose between a safe and a risky decision throughout a game or event [4].

The correlation between sleep and creativity arises from the direct impact of sleep on the process of learning and the development of novel concepts, ideas, or solutions. Research indicates that sleep enhances cognitive abilities for more challenging problem-solving tasks. Elite athletes rely on creative problem-solving as a crucial skill. During every game or competition, professional athletes have choices that might either enhance or diminish their likelihood of winning [4].

Monitoring

Athletes have poor self-assessment of their sleep requirements, duration, and quality, which may decrease their inclination to seek assistance or medical intervention when necessary [3]. Monitoring sleep in athletes can be beneficial for identifying and addressing any decline in performance and a notable decrease in overall health (Table 3) [2].

**Table 3:** Monitoring Techniques and tools for assessing sleep quality and patterns in athletes to identify issues and improve performance and well-being.

Method	Description	References
Hooper index	Subjective wellness questionnaire assessing fatigue, stress, and sleep quality on a 1-7 scale.	Cook and Charest, 2023 [1]
Actigraphy	Non-invasive wearable devices that track movement to estimate sleep duration and quality.	Charest and Grandner, 2020 [4]
Sleep questionnaires	Cost-efficient self-reporting tools, such as the Pittsburgh Sleep Quality Index, to assess sleep patterns and quality.	Copenhaver and Diamond, 2017 [9]
Polysomnography	Gold-standard technique for detailed sleep quality analysis but less practical for athletes.	Watson, 2017 [3]
Sleep applications	Smartphone apps offer convenience but require further validation for reliability in athletic populations.	Bonnar et al., 2018 [15]

Sleep questionnaires are a common and cost-efficient approach to assessing sleep patterns during training and competitive phases. Self-reporting tools allow athletes to introspect and identify specific areas of concern, although they are inherently subjective. A previous study on SD and strategies to increase sleep duration (referred to as sleep extension) recommended using specific questionnaires that focus on different components of sleep, such as sleep disorders, total sleep duration (TSD), bedtime, and wake-up time. The term "sleep extension" specifically refers to the practice of increasing the length of sleep beyond an individual’s usual duration, typically to enhance recovery and performance [2].

The Hooper Index is a questionnaire that assesses sleep quality and overall wellness by evaluating four key parameters: fatigue, stress, delayed-onset physical soreness, and sleep quality. Prior to a training or match session, participants complete the questionnaire based on their emotions and perceptions of the previous training day. The sleep quality question uses a subjective rating scale ranging from 1 to 7 arbitrary units, with 1 indicating extremely poor sleep quality and 7 indicating exceptionally good sleep quality. The scores for the four parameters can also be summed to provide Hooper's overall wellness score [2].

Polysomnography is a benchmark technique that provides information on various aspects of sleep quality and quantity. Given the imperative nature of sleep monitoring, this method requires profound expertise and is not particularly beneficial for athletes [2].

Actigraphy, an alternate technique, utilizes watches equipped with motion-tracking sensors to monitor body movement. By combining this data with sleep logs, octopography may reliably determine sleep length, startle latency, wake-up time, and sleep quality. This method effectively assesses sleep, is non-intrusive, and can collect data for a duration of two weeks [2].

The utilization of smartphone sleep applications may be contemplated due to the ease of integration it may provide in athletes' lives; however, additional testing is still necessary to ascertain the validity and reliability of such gadgets and apps [2].

Interventions

In response to the increasing concern regarding athletes' sleep, there has been a significant rise in the number of studies investigating sleep interventions in recent years (Table 4).

**Table 4:** Interventions Evidence-based strategies, including behavioral, environmental, and pharmacological approaches, to enhance sleep quality and duration for athletes.

Intervention	Description	References
Bright light therapy	Used to adjust circadian rhythms and improve alertness during jet lag or irregular schedules.	Halson and Juliff, 2017 [6]
Total sleep duration	Extending nighttime sleep or incorporating naps (20–90 minutes) improves physical and cognitive performance, mood, and recovery.	Cunha et al., 2023 [7]
Pharmacological interventions	Use of melatonin or sedative-hypnotics (e.g., zolpidem, eszopiclone) for short-term sleep adjustment; benzodiazepines are discouraged due to dependency risks.	Holgado et al., 2022 [17]
Post-exercise recovery strategies	Techniques such as whole-body cryostimulation enhance sleep onset and efficiency and aid physiological recovery, and red light therapy enhances sleep quality.	Altarriba-Bartes et al., 2020 [16]
Sleep hygiene	Techniques like maintaining a dark, quiet bedroom environment, reducing electronic device usage, and establishing wind-down routines improve sleep quality.	Bonnar et al., 2018 [15]
Education programs	Tailored sleep education for athletes and coaches to address sleep patterns, the importance of recovery, and practical strategies for better sleep.	Bonnar et al., 2018 [15]

Total Sleep duration and Napping

According to a systematic review conducted by Cunha et al., increasing the TSD by taking naps or sleeping longer at night can positively affect physical and/or cognitive function. For athletes who consistently get around seven hours of sleep every night, the recommendation could be to increase their sleep length by up to two hours or by taking a nap during the day. lasting between 20 and 90 minutes [7]. Increasing the TSD athletes acquire seems to enhance their ability to perform sports-specific skills and cognitive tasks, such as reaction time and shooting accuracy. Naps increase TSD, improve performance after a normal night's sleep, and restore performance to baseline after partial SR [7].

Additionally, with the increase in TSD, there were positive outcomes in terms of enhanced mood and decreased daily drowsiness. This discovery aligns with studies on partial SD, which suggest that it negatively affects cognitive abilities and emotions, perhaps resulting in worse overall performance [15].

Sleep Hygiene

Strategies including sleep hygiene, mindfulness, and reducing electronic device usage before bedtime may positively impact performance outcomes by improving sleep quality and/or length. Utilizing light exposure strategies can be a viable approach to altering the circadian rhythm and enhancing the vigilance of athletes during periods of declining alertness, such as at night [7].

Implementing acute sleep hygiene measures may lead to an increase in the amount of sleep athletes get but does not have a significant impact on their subsequent performance and recovery. On the other hand, implementing long-term sleep hygiene may be more advantageous [15].

To mitigate the adverse consequences of jet lag, a combination of sleep hygiene and bright light therapy can be useful [15].

Post-exercise Recovery Strategies to Improve Nocturnal Sleep

Bonnar et al. systematically reviewed post-training recovery tactics designed to induce advantageous physiological responses regarding recovery, sleep initiation, and maintenance. Examples of these effects include improved parasympathetic reactivation [15].

Whole-body cryostimulation enhances many sleep metrics, including sleep length, onset, and efficiency, but it doesn’t seem to impact sleep quality. It also improved the physiological response to exercise, including decreased blood lactate levels, heart rate, and alpha-amylase, while reducing physical tiredness [16].

Exposure to red light at night revealed a beneficial impact on both subjective and objective sleep patterns and slight enhancements in performance assessments. After 30 minutes of red-light exposure at night, participants exhibited enhanced subjective sleep quality as measured by the PSQI. Additionally, their morning serum melatonin levels were higher, suggesting a circadian delay caused by the red light. In terms of performance, participants in the intervention group demonstrated a clear improvement in the long-distance running test, as measured by the Cooper 12-minute run [15].

Education

A sleep education program designed for athletes should focus on assessing sleep patterns, identifying common sleep-related issues, emphasizing sleep's vital role in optimizing performance and recovery, and providing strategies to improve overall sleep quality. Clear and realistic expectations about sleep should be established to prevent undue stress related to variations in sleep patterns. To increase engagement and relevance, the program should be customized to the specific demands of each sport, ensuring it resonates with athletes and fosters their active participation [15].

Behavioral Strategies

Collaborating with athletes and coaches to develop strategies for increasing sleep opportunities can involve practical adjustments, such as delaying morning training sessions by 30 minutes to allow for additional morning sleep or encouraging earlier bedtimes. If extending nighttime sleep is not feasible, incorporating a nap at least two hours after training could be particularly beneficial, as it helps restore energy and enhances readiness for a second training session later in the day [15].

It is important to limit sleeping in on weekends to within one hour of the usual weekday wake-up time, even when it allows for longer sleep. Engaging in prolonged sleep in the morning reduces the want to sleep, making it harder to fall asleep at the normal time the next night. It also prevents exposure to bright morning light, which helps regulate the body's internal clock [15].

Effective sleep hygiene entails implementing simple techniques, such as ensuring that the bedroom environment is at a comfortable temperature, dark, and quiet (e.g., by providing earplugs and eye masks), limiting physically, cognitively, and emotionally stimulating activities before going to bed (such as scheduling wind-down time after late-night games), and utilizing dim lighting up to two hours prior to bedtime [16].

Based on the research included in the current review, consistently implementing these approaches for a period of one to three months is likely to lead to improvements in sleep and performance [15].

Pharmacology Interventions

Nonpharmacological approaches are the first-line treatment for sleep problems in athletes. However, these approaches can be unfeasible for athletes with demanding schedules and limited time availability. Hence, the utilization of specific drugs can assist athletes in expediting the adjustment of their sleep patterns while maintaining optimal performance levels. The existing body of research indicates that the use of melatonin and sedative-hypnotic sleep aids can effectively facilitate the alteration of the sleep cycle [17].

Melatonin has been extensively researched as a beneficial agent for both athletes and nonathletes who require sleep phase advancement. Short-term declines in physical performance are observed shortly following consumption; hence, it is advisable to provide a period of eight hours of sleep prior to engaging in sports activities [17].

Sedative-hypnotic sleep aids, such as zolpidem and eszopiclone, promote the advancement of the sleep phase, functioning as secondary options to melatonin. The enhancement of sleep quality renders them appealing to sportsmen during travel and the nocturnal period preceding competitions [17].

Short-acting medicines such as benzodiazepines are commonly used in conjunction with an eight-hour sleep period preceding competition to mitigate the occurrence of "hangover" effects and minimize any detrimental impact on performance. The avoidance of benzodiazepines is advocated, considering their propensity for abuse and the availability of more effective alternatives [17].

## Conclusions

Sleep plays a crucial role in the overall well-being and performance of athletes. Inadequate sleep can negatively affect physical performance, cognitive function, and mental health. Athletes often experience poor sleep quality and insufficient sleep duration, which can be attributed to competition-related stress, travel, and training schedules. Lack of sleep can lead to decreased athletic performance, increased risk of injuries, and impaired recovery. Sleep tracking can be beneficial in diagnosing and treating sleep issues in athletes. Strategies to improve athlete sleep include increasing total sleep duration, implementing sleep hygiene practices, and utilizing pharmacological interventions when necessary. Practices such as increasing sleep duration through naps or longer nighttime sleep, mindfulness, good sleep hygiene, and reducing screen time before bed may also positively affect performance outcomes. This study has limitations, including variability in methodologies, sample sizes, participant demographics, and sleep measurement techniques, which hinder generalization. Many studies rely on subjective self-reported data prone to bias, and there is a lack of research on the long-term effects of sleep interventions in athletes. Additionally, the interaction between factors like age, sport type, and training intensity and their impact on sleep remains insufficiently explored. Further research is needed to validate these findings and improve the data quality in this area. Studying the impact of sleep restriction on both physical and cognitive performance would be of interest since this is a common scenario in athletics. It is important for athletes and coaches to prioritize sleep and incorporate sleep education and interventions into training programs to optimize performance and overall health.
